# ^17^O-Dynamic NMR and DFT Investigation of Bis(acyloxy)iodoarenes

**DOI:** 10.3390/molecules171112718

**Published:** 2012-10-26

**Authors:** Luca Fusaro, Francesca Mocci, Michel Luhmer, Giovanni Cerioni

**Affiliations:** 1 Laboratoire de RMN Haute Résolution CP 160/08, Université Libre de Bruxelles, Av. F.-D. Roosevelt 50, 1050 Brussels, Belgium; Email: lfusaro@ulb.ac.be (L.F.); mluhmer@ulb.ac.be (M.L.); 2 Dipartimento di Scienze Chimiche e Geologiche, Università di Cagliari, Complesso Universitario, S.S. 554, Bivio per Sestu, I-09042 Monserrato (CA), Italy

**Keywords:** hypervalent iodine, fluxional compounds, sigmatropic shift, ^17^O-NMR, chemical exchange, Density Functional Theory

## Abstract

Bis(acetoxy)iodobenzene and related acyloxy derivatives of hypervalent I(III) were studied by variable temperature solution-state ^17^O-NMR and DFT calculations. The ^17^O-NMR spectra reveal a dynamic process that interchanges the oxygen atoms of the acyloxy groups. For the first time, coalescence events could be detected for such compounds, allowing the determination of activation free energy data which are found to range between 44 and 47 kJ/mol. The analysis of the ^17^O linewidth measured for bis(acetoxy)iodobenzene indicates that the activation entropy is negligible. DFT calculations show that the oxygen atom exchange arises as a consequence of the [1,3]-sigmatropic shift of iodine. The calculated activation barriers are in excellent agreement with the experimental results. Both the ^17^O-NMR and DFT studies show that the solvent and chemical alterations, such as modification of the acyl groups or *para-* substitution of the benzene ring, hardly affect the energetics of the dynamic process. The low I-O Wiberg bond index (0.41–0.42) indicates a possible explanation of the invariance of both the energy barrier and the ^17^O chemical shift with *para-*substitution.

## 1. Introduction

**Figure 1:**
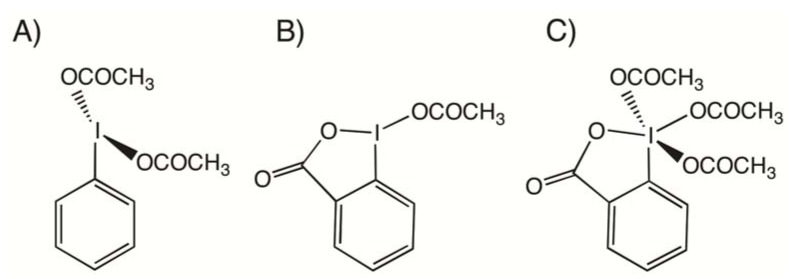
Structure of (**A**) bis(acetoxy)iodobenzene, (**B**) (acetoxy)benziodoxolone and (**C**) the Dess-Martin periodinane.

Hypervalent iodine chemistry is a field of great interest as shown by the large body of literature dealing with its various aspects [1,2,3,4,5,6]. For some years our group has been interested in studying the I-O bond and the dynamical behavior of some important I(III) and I(V) organic derivatives, such as bis(acyloxy)iodoarenes, benziodoxolones and the Dess-Martin periodinane (Figure 1), by combining solution-state ^17^O-NMR and Density Functional Theory (DFT) calculations [7,8,9]. 

**Figure 2:**
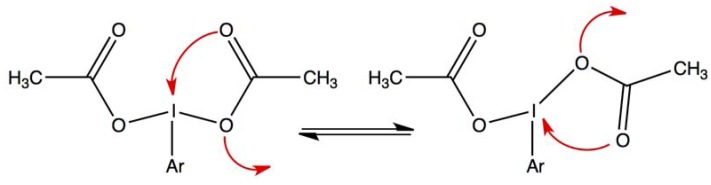
Schematic representation of the [1,3]-sigmatropic shift of iodine in bis(acetoxy)iodoarenes.

At room temperature, the ^17^O-NMR spectrum of all the studied bis(acyloxy)iodoarenes showed a single signal for both the carbonylic (O_1_) and ester-like (O_2_) oxygen atoms of the carboxylic groups. Similarly, a single signal was observed for the lateral acetoxy groups of the Dess-Martin reagent and, on the other hand, for the central acetoxy group of this λ^5^ iodane. With benziodoxolones, in contrast, distinctive ^17^O-NMR signals were observed at 25 °C for the O_1_ and O_2_ atoms of the carboxylic group not involved in the iodoxolone ring. However, at 45 °C, these two signals could not be detected. DFT calculations revealed that, among the various possible explanations, the [1,3]-sigmatropic shift of iodine between the two oxygen atoms of the carboxylic residue(s) (see Figure 2) is the actual cause of the experimental observations. The corresponding activation barriers were calculated and found to be in agreement with the ^17^O-NMR results. Summing up, our previous studies showed that the I-O bond of either I(III) or I(V) acyloxy derivatives is fluxional as a consequence of a [1,3]-sigmatropic shift mechanism. The activation barrier is much higher in the studied λ^3^ iodane than in the Dess-Martin λ^5^ iodane. Furthermore, in both the I(III) and I(V) acetoxy derivatives, the barrier is higher if the I-O bond is coplanar with the aromatic ring. The energetics and, consequently, the kinetics of the degenerate [1,3]-sigmatropic shift of iodine are thus affected by the environment of the carboxylic group. On the other hand, in a series of *para*-substituted bis(acetoxy)iodobenzenes, we observed a remarkable invariance of the ^13^C and ^17^O chemical shifts of the acetoxy groups. This is not yet understood. Indeed, the hypothesis of tight ion pairs that was first considered [7] is invalidated by our subsequent studies summarized above [8,9].

Dynamic NMR (DNMR) of low-sensitivity and fast-relaxing nuclei, among which is ^17^O, is definitely not routine in the study of organic molecules. Indeed, near the coalescence temperature, baseline distortion and poor signal-to-noise ratio may prevent the acquisition of NMR spectra that are suitable for line shape analysis while, in the intermediate-slow or -fast exchange regime, the natural linewitdh is not negligible and, moreover, may show a strong temperature dependence. Actually, coalescence could not be observed in any of our previous ^17^O-NMR studies of hypervalent iodine compounds. The acyloxy signal(s) of the I(III) derivatives, for which the coalescence temperature is accessible to solution-state NMR, broadened beyond detection as a consequence of the chemical exchange process itself or, at low temperature, as a consequence of quadrupole relaxation. Only rough estimations of the activation free energy (ΔG^#^) could therefore be provided. Recently, using acetoxysilanes as model compounds, we showed that currently available NMR instrumentations and pulse sequences allow for quantitative ^17^O-DNMR studies to be carried out without enrichment being needed [10]. This opened the perspective to precisely determine ΔG^#^ values for the [1,3]-sigmatropic shift of iodine in I(III) acyloxy derivatives and, also, to determine the activation enthalpy (ΔH^#^) and the activation entropy (ΔS^#^) of this dynamic process. It is noteworthy that, in acetoxysilanes of the general formula (CH_3_COO)_n_Si(CH_3_)_4-n_ with n = 1–4, the [1,3]-sigmatropic shift of the silicon atom is characterized by a significant activation entropy [10].

**Figure 3:**
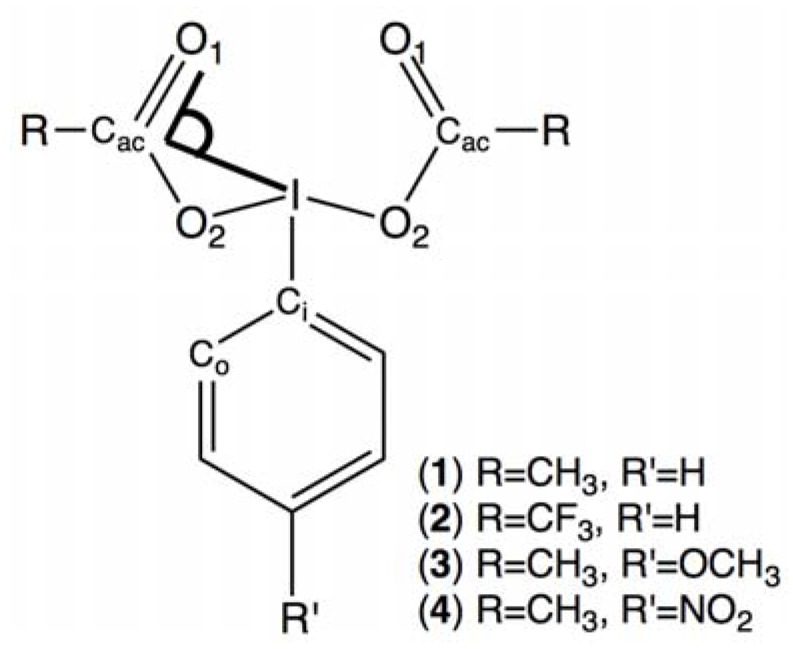
Studied compounds with atom labeling and the angle I-C_ac_-O_1_ in bold.

The present work reports on several bis(acyloxy)iodoarenes: bis(acetoxy)iodobenzene (**1**), bis(trifluoroacetoxy)iodobenzene (**2**), *para*-methoxy-bis(acetoxy)iodobenzene (**3**) and *para*-nitro-bis(acetoxy)iodobenzene (**4**) (Figure 3). These λ^3^ iodanes were studied by ^17^O-DNMR with the primary goal of investigating the effect of electron-withdrawing and electron-donating groups on the energetics of the [1,3]-sigmatropic shift of iodine. Possible solvent effects were also investigated. The experimental data are compared to the results of DFT calculations.

## 2. Results and Discussion

### 2.1. Bis(acetoxy)iodobenzene (***1***)

**Figure 4:**
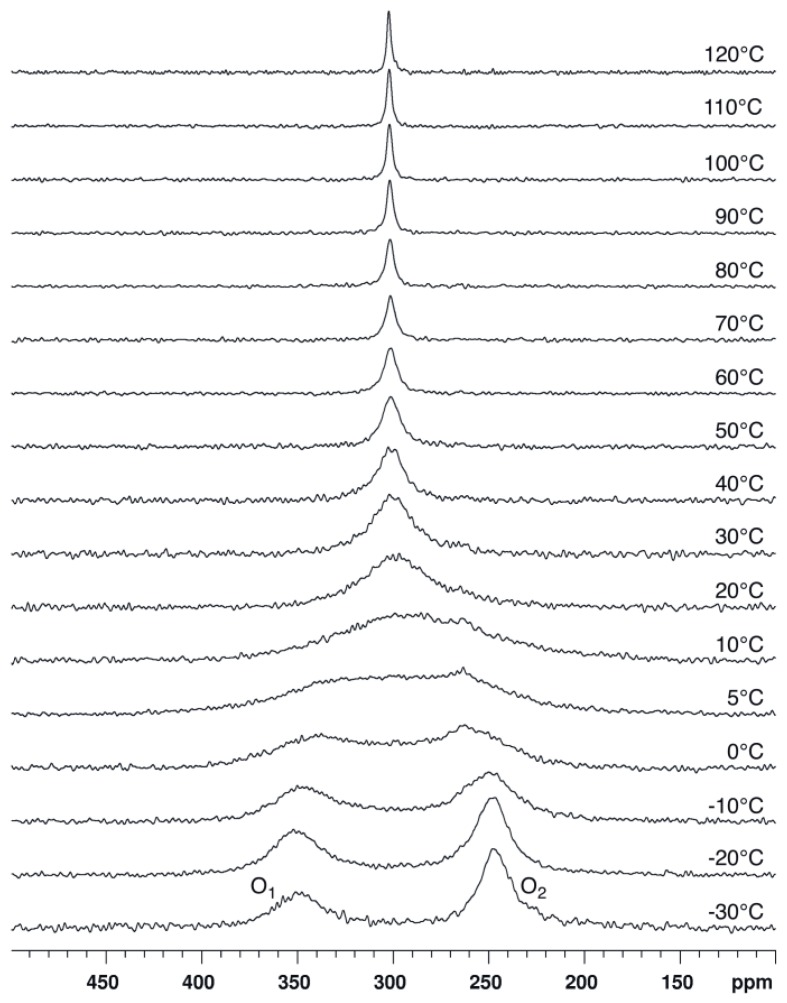
^17^O-NMR spectra recorded at 14.1 T and various temperatures for compound **1** dissolved in CDCl_3_. No apodization of the FID was used before Fourier transform.

^17^O-NMR spectra of compound **1** dissolved in chloroform-*d_1_* (CDCl_3_) were recorded at 14.1 T and various temperatures ranging between −30 and 110 °C (Figure 4). Two signals of similar integrated intensity are observed at low temperature (see Supporting Information). On the basis of chemical shift data reported for similar compounds [8,9,10,11,12], the signal at about 350 ppm is assigned to the carbonylic O_1_ oxygen atoms and, conversely, the signal at about 250 ppm is assigned to the ester-like O_2_ oxygen atoms. At −30 °C, the chemical shift (δ) and the full linewidth at half height (LW), as determined by fitting Lorentzian lines, are respectively 349 ppm and 3.28 kHz for the O_1_ signal and 246 ppm and 1.49 kHz for the O_2_ signal. Both these signals broaden for increasing temperature and coalescence occurs at about 5 °C. At 10 °C, a single signal is observed at δ ≈ 300 ppm, *i.e.*, at the average of the O_1_ and O_2_ chemical shift values measured at low temperature. The LW of this signal decreases for increasing temperatures while the chemical shift is essentially constant.

These observations are consistent with the evolution expected for an equally populated two-site exchange. At the coalescence temperature (T_c_), the first order rate constant characterizing such an exchange process is directly related to the difference in resonance frequency in each site (Δν = ν_1_ ‑ ν_2_); the activation free energy at T_c_ can then be determined via the Eyring equation (see Supporting Information). For a chemical shift difference Δδ = (δ_1_ − δ_2_) = 103 ppm (Δν = 8.30 kHz at 14.1 T) and T_c_ = 5 °C, ΔG^#^ is estimated to be (45.2 ± 0.5) kJ/mol. The error on ΔG^#^ was estimated considering an absolute error on T_c_ of ± 3 °C. It is noteworthy that an error of ±5 ppm on Δδ affects ΔG^#^ by only ±0.1 kJ mol^−1^.

**Figure 5:**
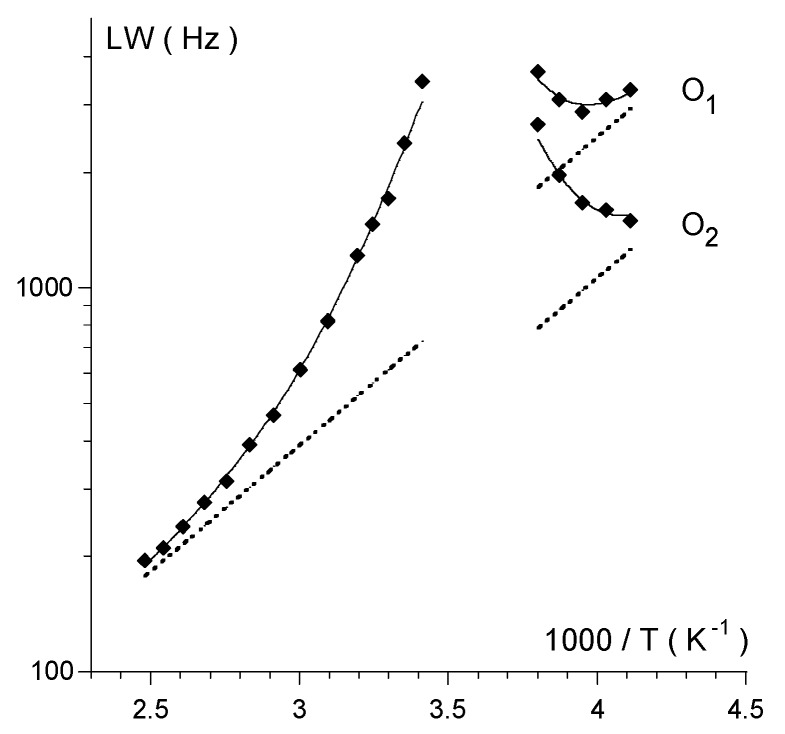
Full linewidth at half height (LW) measured at 14.1 T for the ^17^O-NMR signal(s) of compound **1** dissolved in CDCl_3_. The LW data were determined by fitting two Lorentzian lines of equal integrated intensity to the experimental spectra recorded at T ≤ −10 °C and one single Lorentzian line at T ≥ 20 °C. The solid lines are the results of the best fit analysis according to Equations (1)–(4). The natural linewidth contributions (shown as dashed lines) are characterized by the following parameters: E_q_ = (12.6 ± 0.2) kJ/mol, LW_q_^298^ = (0.94 ± 0.02) kHz and (0.40 ± 0.02) kHz for O_1_ and O_2_, respectively [14].

Further information can be gained from the temperature dependence of the LW, which is shown in Figure 5 and was analyzed according to Equations (1–4), as in our previous ^17^O-DNMR investigation [10].


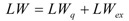
(1)


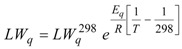
(2)


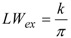
(3a)


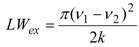
(3b)


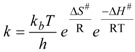
(4)

In Equation (1), LW_q_ is the natural linewidth due to quadrupole relaxation and LW_ex_ is the contribution due to chemical exchange. The temperature dependence of LW_q_ is accounted for by an Arrhenius-type expression (Equation (2)) where the pre-exponential factor is the natural linewidth at 298 K and E_q_ is the activation energy. Identical activation energy was assumed for the quadrupole relaxation of both the O_1_ and O_2_ oxygen nuclei. Above the coalescence temperature, LW_q_ is given by the arithmetic average of the natural linewidth values predicted for O_1_ and O_2_. The broadening due to chemical exchange between two equally populated sites is given by Equations (3a) or (3b), depending on whether the kinetics is slow or fast on the NMR spectral time scale. In Equation (3b), ν_1_− ν_2_ is the frequency difference between the O_1_ and O_2_ signals, k is the first-order rate constant, the temperature dependence of which is given by the Eyring equation (Equation (4)). ΔS^#^ and ΔH^#^ are the activation entropy and the activation enthalpy, respectively, which are assumed to be temperature independent, and the other symbols have their usual meaning. The best-fit of Equations (1)–(4) to the experimental LW data, using a least-squares technique based on the relative residuals [13], yields ΔG^#^ = (45.5 ± 0.1) kJ/mol at 25 °C, which agrees with the value estimated at the coalescence (*vide supra*), ΔH^#^ = (45 ± 1) kJ/mol and ΔS^#^ = (−1 ± 5) J mol^−1^ K^−1^ [14]. Interestingly, the LW analysis reveals that the entropic contribution is not significant.

**Figure 6:**
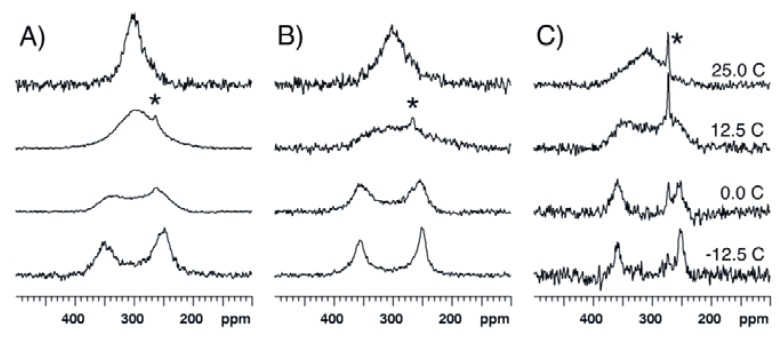
^17^O-NMR spectra recorded at 14.1 T and various temperatures for compound **1** dissolved in (**A**) CDCl_3_, (**B**) CD_2_Cl_2_ and (**C**) CD_3_CN. The asterisk indicates the peak of acetic acid. The spectra recorded at low temperature in CD_3_CN exhibit a low signal-to-noise ratio as a consequence of poor solubility of **1**.

In order to investigate possible solvent effects, ^17^O-NMR spectra were recorded at 14.1 T and several temperatures for compound **1** dissolved in dichloromethane-*d_2_* (CD_2_Cl_2_) and in acetonitrile-*d_3_* (CD_3_CN). The spectra recorded in these more polar solvents are highly similar to the spectra recorded at the same temperature in CDCl_3_ (Figure 6). T_c_ is found to be about 10 °C in CD_2_Cl_2_ and is somewhat higher, ~15 °C, in CD_3_CN. Similar chemical shift values are observed, leading to similar ΔG^#^ values (Table 1).

### 2.2. Bis(trifluoroacetoxy)iodobenzene (***2***)

**Figure 7:**
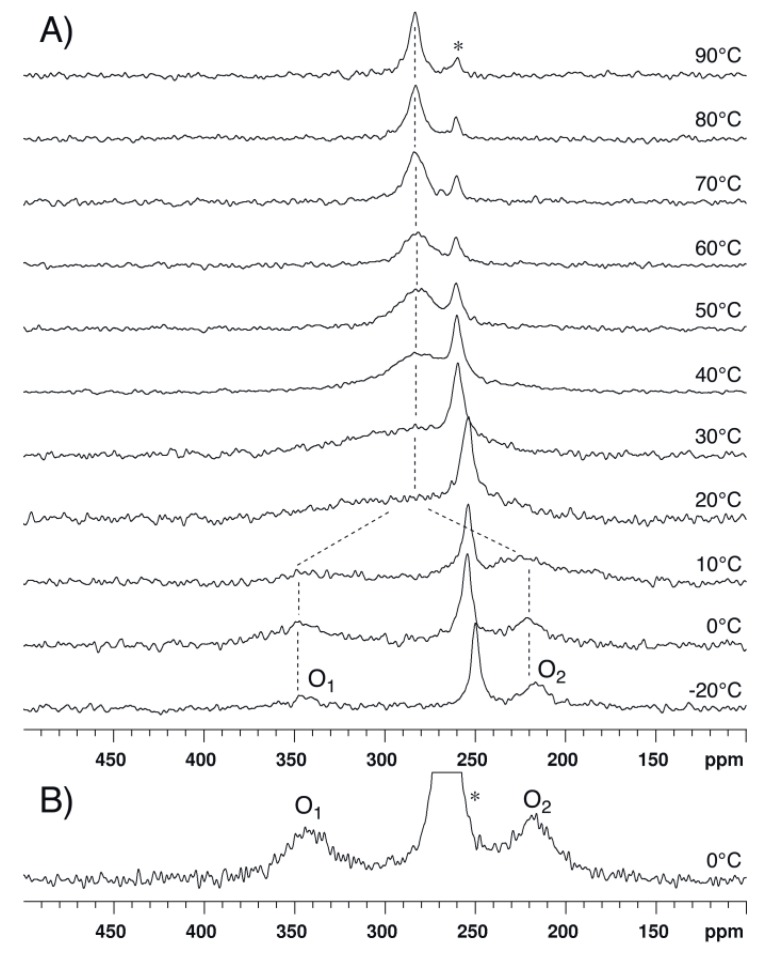
^17^O-NMR spectra recorded at 14.1 T for compound **2** dissolved in (**A**) CDCl_3_ and (**B**) CD_3_CN. An exponential multiplication of the FID with a line broadening factor of 100 Hz was used before Fourier transform. The dotted lines are guides for the eyes. The asterisk indicates the peak of trifluoroacetic acid.

^17^O-NMR spectra of compound **2** dissolved in CDCl_3_ were recorded at 14.1 T and various temperatures ranging between −20 and 90 °C (Figure 7A). At low temperature, the signal of trifluoroacetic acid, which is present as an impurity in the sample, is the most intense signal because **2** is not entirely soluble and also because the ^17^O-NMR signals of **2**, which could be detected at about 350 ppm (O_1_) and 220 ppm (O_2_), are highly broadened. At 0 °C in CD_3_CN (Figure 7B), the O_1_ and O_2_ signals are observed at 341 ppm and 218 ppm, respectively. Similarly to the spectra of compound **1**, the spectra of **2** reveal the occurrence of a dynamic process which interchanges O_1_ and O_2_. The coalescence is observed at about 20 °C in CDCl_3_ and ΔG^#^ is estimated to be (47.3 ± 0.5) kJ/mol.

### 2.3. para-Methoxy-Bis(acetoxy)iodobenzene (***3***) and para-Nitro-Bis(acetoxy)iodobenzene (***4***)

**Figure 8:**
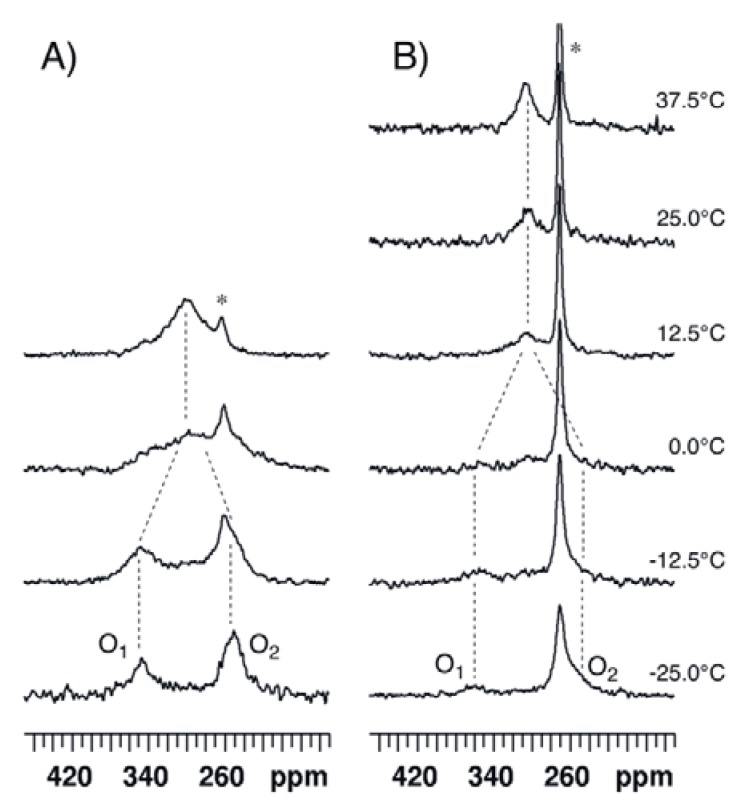
^17^O-NMR spectra recorded at 14.1 T and various temperatures (**A**) for compound **3** dissolved in CDCl_3_ and (**B**) for compound **4** dissolved in CDCl_3_/DMSO-d_6_ 98/2. An exponential multiplication of the FID with a line broadening factor of 100 Hz was used before Fourier transform. The dotted lines are guides for the eyes. The asterisk indicates the peak of acetic acid.

^17^O-NMR spectra of compound **3** dissolved in CDCl_3_ were recorded at 14.1 T and various temperatures ranging between −25 and 12.5 °C (Figure 8A). The ^17^O-NMR signal of the methoxy group was detected at 62 ppm, *i.e.*, in the expected range of chemical shift [15] (see Supporting Information). At −25 °C, the O_1_ and O_2_ signals are observed at 349 ppm and 254 ppm, respectively. The coalescence occurs somewhat below 0 °C; using T_c_ = −5 °C, ΔG^#^ is estimated to be 43.7 kJ/mol. 

For solubility reasons, compound **4** was dissolved in a 98/2 (v/v) mixture of CDCl_3_ and DMSO-*d_6_* rather than in pure CDCl_3_. The ^17^O-NMR spectra were recorded at 14.1 T and various temperatures ranging between −25 and 37.5 °C (Figure 8B). The signal of the NO_2_ group was observed at 573 ppm, *i.e.*, in the expected range of chemical shift [15] (see Supporting Information). At −25 °C, the O_1_ signal is observed at 359 ppm. The solubility of **4** is rather low and, consequently, acetic acid gives rise to an intense signal which masks the O_2_ signal at about 255 ppm. Coalescence is observed between 0 and 12.5 °C; using T_c_ = 5 °C, ΔG^#^ is estimated to be 45.2 kJ/mol. 

**Table 1:** Activation barriers calculated at the PBE0/LANL2DZDP level and summary of the experimental results for compounds **1**–**4**. Unless otherwise stated, the activation barrier (ΔE) was computed *in-vacuo*, the chemical shift of the O_1_ and O_2_ signals (δ_1_ and δ_2_) were determined by fitting Lorentzian lines to the spectrum recorded at the lowest investigated temperature, the activation free energy (ΔG^#^) was determined at the coalescence temperature (T_c_) and the error on ΔG^#^ is estimated to be ±0.5 kJ mol^−1^. This error is due to the error on T_c_, which is estimated to be ± 3 °C. n.d.: not determined.

	ΔE	Solvent	δ_1_	δ_2_	Δδ	T_c_	ΔG^#^
	kJ mol^−^^1^		(ppm)	(ppm)	(ppm)	(°C)	kJ mol^−^^1^
**1**	45.2 48.3 ^a^	CDCl_3_	349	246	103	5	45.5 ± 0.1 ^b^
		CD_2_Cl_2_	355	251	104	10	46.0
		CD_3_CN	358	251	107	15	46.8
**2**	48.4	CDCl_3_	345 ^c^	221 ^c^	124	20	47.3
		CD_3_CN	341	218	123	n.d.	n.d.
**3**	44.4	CDCl_3_	349	254	95	−5	43.7
**4**	45.2	CDCl_3_/DMSO-*d_6_*^d^	359	~255 ^e^	~104	5	45.2

^a^ Computed in CHCl_3_ described as a polarizable continuum. ^b^ Value at 25 °C determined by the analysis of the linewidth data. The value estimated at the coalescence temperature is 45.2 kJ mol^−1^. ^c^ Estimated on the spectrum recorded at 0 °C. ^d^ 98/2 (v/v). ^e^ Estimated on the spectrum without fitting.

### 2.4. Comparisons with DFT Calculations and Discussion

Table 1 shows that the chemical shift of O_1_ is barely affected by the trifluoromethyl group but the chemical shift of O_2_ is reduced by about 30 ppm with respect to the value measured for bis(acetoxy)iodobenzene **1**. In contrast, the ^17^O chemical shift data are quite insensitive to the *para-*substitution of the aromatic ring. This confirms the previous observations made for the average signal detected in the fast exchange regime [7]. In the present work, the O_1_ and O_2_ signals are, for the first time, distinctively observed and it can now be stressed that the previous observations are not the consequence of opposite chemical shift variations. 

The ^17^O-DNMR results obtained for compound **1** indicate that, in CDCl_3_, the activation entropy characterizing the oxygen atom exchange is negligible and suggest that the activation free energy, about 46 kJ/mol, is rather insensitive to the dielectric constant of the solvent. The ^17^O-DNMR results also indicate that ΔG^#^ is hardly affected by the chemical alteration present in compounds **2**–**4**. Indeed, the ΔG^#^ data measured for **4** and **1** are not significantly different. The replacement of the acetoxy groups of **1** by trifluoroacetoxy groups increases ΔG^#^ by less than 2 kJ/mol while a methoxy group in *para-*position has the opposite effect.

As mentioned in the Introduction, the oxygen atom exchange in **1** was ascribed to a [1,3]-sigmatropic shift of iodine [8]. The activation energy barrier (ΔE) of this process can be calculated as the total energy difference between the transition state (TS) and the most stable rotamer. Our previous DFT study of compound **1** yielded ΔE = 45.2 kJ/mol *in vacuo* [8], a value which is in excellent agreement with the experimental results (Table 1). In our previous study on **1** and related compounds, we observed that the calculated energy profiles do not vary substantially when including the solvent, which was described in the framework of the polarizable continuum model (PCM) [16,17,18,19]. The DFT calculations yielded ΔE = 48.3 kJ/mol for **1** in chloroform. No important solvent effect on the activation energy barrier was observed and this also agrees with the experimental results of this work. On the base of these considerations, we believe that *in vacuo* calculations provide reliable results for the systems under investigations. In principle, it is the calculated activation free energy, and not ΔE, which should be compared to the experimental ΔG^#^ values. However, various comparisons of DFT and experimental results have shown that the best agreement is obtained when no thermodynamical correction is applied to the calculated total energy; this point is discussed in the recent review of Casarini *et al*. [20]. Reasonably, the comparison between calculated energy barriers and experimental activation free energies is meaningful if the activation entropy is negligible or small, as it is the case for compound **1**. In the following, it is assumed that there is no significant entropic contribution to the activation free energy measured for compounds **2**–**4**. 

**Figure 9:**
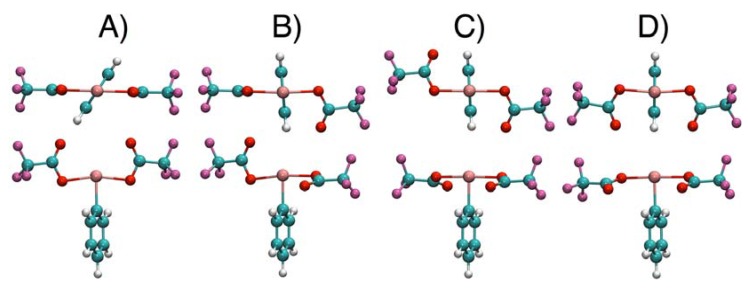
Optimized Pbe0/LANL2DZDP stable conformations of compound **2**. Two representations are given for each conformer; they are rotated by approximately 90° around the I-O bonds. Energy of each conformer is given in Table 2 using the same label (A, B, C or D) as in the figure. Color code: cyan = carbon, white = hydrogen, red = oxygen, violet = fluorine, pink = iodine.

DFT calculations were completed for compounds **2**–**4** with the main objective of verifying if the interchange of O_1_ and O_2_ observed by NMR for these λ^3^ iodanes can also be explained by the [1,3]-sigmatropic shift of iodine. The minimum energy structures of **2**–**4** show strong resemblance with those of **1** (see Figure 9 for **2**), although some differences in the relative energies can be noted (Table 2). The stable rotamers of **2** exhibit the smallest variations in total energy with essentially the same value for the conformations A and B.

**Table 2:** Relative total PBE0/LANL2DZDP energy (kJ/mol) of the stable rotamers of compounds **1**-**4**. Labels indicating the rotamers as in Figure 9.

Conformer	1	2	3	4
***A***	0.0	0.4	0.0	0.0
***B***	1.7	0.0	0.8	3.7
***C***	5.7	1.6	8.4	8.1
***D***	9.3	3.2	7.6	7.7

Compound **2** is the one showing the largest experimental differences with compound **1**. To verify whether the differences might be related to differences in the exchange mechanism, the latter was studied as previously done for compound 1 in reference [8]. The calculated energy profile is reported in Table 3 together with relevant structural data. It is similar to the profile found for **1**, in having the maximum for a I-C_ac_-O_1_ angle comprised between 55° and 60° and in involving a rotation of 90° of the carboxylate plane during the shift (in this way going from conformation **A** to **B**).

It is reasonable to assume that the same exchange mechanism occurs for compound **3** and **4**. In all cases, the calculated TS (see Supporting Information) are similar to that of **1** and the normal mode corresponding to the imaginary frequency clearly involves the oxygen atoms exchange. The energy barriers calculated in vacuo for compounds **2**–**4** are given in Table 1. The agreement with the corresponding experimental activation free energy is excellent, indicating that all of the studied bis(acyloxy)iodobenzenes are fluxional as a consequence of the [1,3]-sigmatropic shift of iodine and corroborating that this process is indeed quite insensitive to chemical alterations such as para- substitution of the arene moiety or modification of the acyl groups.

**Table 3:** Selected dihedrals and PBE0/LANL2DZDP total energy differences, calculated *in vacuo* with respect to the most stable rotamer, at variable I-C_ac_-O_1_ angle for compound **2**. Atom labeling as in Figure 3.

I-C_ac_-O_1_ (°)	ΔE kJ/mol	Dihedral angles (°)	
		C_i_-I-O_1_-C_ac_	C_o_-C_i_-I-O_2_^a^
80	0.6	0.2	58.5
75	3.4	1.3	62.3
70	10.2	0.0	90.0
65	22.0	0.0	90.0
60	38.3	0.0	90.0
55	26.3	−78.2	90.0
50	13.5	−78.5	90.1
45	3.3	−77.9	92.1
40	0.0	−76.7	96.3
TS (58.3)	48.4	−41.9	76.5

^a^ O_2_ belongs to the unconstrained moiety. For a direct comparison among the dihedrals of the considered atomic configurations of **1** reported in a previous paper [8], the conformational enantiomers with the smallest C_o_-C_i_-I-O_2_ angle between the unconstrained acetoxy unit and the clockwise C_o_ were considered (view along the I-C_i_ bond).

As mentioned in the Introduction, *para-*substitution of bis(acetoxy)iodobenzenes does not significantly affect the ^13^C nor ^17^O chemical shift of the acetoxy groups [7]. This contrasts with the observations on relatively similar systems, such as *para*-substituted benzyl alcohols [21] and phenylacetic acids [22]. We are not able, for the moment, to give a clear-cut explanation of this difference but the lack of electronic effect transmission between iodine and oxygen might be related to the bond order. X-ray measurements [23,24] on compound **1** showed that the I-O bond length is 216 pm, a figure slightly greater than the sum (201.9 pm) of the iodine (134.5 pm) and oxygen (67.4 pm) covalent radii [25], and smaller than the sum (348 pm) of the iodine (196 pm) and oxygen (152 pm) van der Waals radii [26]. Similar results are observed for the I-S distance, as reported by Varvoglis on some carbamoyl(diaryl)iodanes [27,28]. X-ray studies showed this distance to be 305.3 pm, a value comprised between the sum of the iodine and sulphur covalent radii (238.7 pm) and the sum of their van der Waals radii (400 pm). From these data, as well as other significant arguments, the I-S bond order was estimated to be 0.5 [27]. Good estimate of bond orders can be obtained by computation. In fact, Kiprof [29] has shown for a series of hypervalent iodine derivatives that there is a quite good correlation between the experimental I-O bond length and the I-O bond order as estimated by the Wiberg bond index. Kiprof's value of the I-O bond order of **1** is 0.41, in full agreement with the values computed in this work for the minimum energy conformation (Table 4). We note that the I-O bond order, which has been shown [29] to be affected by variation of the substituent at the iodine atom, is actually insensitive to change of the acyl groups or to *para*-substitution of the aromatic ring. It thus seems reasonable that the rather low energy barrier of the [1,3]-sigmatropic shift of iodine in compounds **1**–**4** is related to the low I(III)-O bond order in the stable conformations. Finally, it is noteworthy that the I-O bond order is somehow conserved in the transition state.

**Table 4:** Wiberg bond indices for compounds **1**–**4** calculated at the PBE0/LANL2DZDP level.

Compound	Global Minimum		Transition State	
	I-O_1_	I-O_2_	I-O_1_	I-O_2_
**1**	0.06	0.42	0.28	0.16
**2**	0.04 (0.02) ^a^	0.42 (0.42) ^a^	0.25	0.15
**3**	0.07	0.41	0.27	0.16
**4**	0.08	0.42	0.29	0.15

^a^ Differently from the other compounds the global minimum of **2** is the conformation ***B*** with two non equivalent moieties (see Figure 9). In parenthesis are reported the data for the carboxylate group with the plane perpendicular to the I-C_i_ bond.

## 3. Experimental

Compounds **1** and **2** were purchased from Sigma-Aldrich (Bornem, Belgium) and used as received. Compounds **3** and **4** were synthesized according to a procedure described in literature [30,31]. CDCl_3_, CD_2_Cl_2_, and DMSO-*d_6_* were purchased from Sigma-Aldrich, CD_3_CN was purchased from Euriso-top (Paris, France). The samples used for the ^17^O-NMR measurements were saturated solution prepared at room temperature in 5 mm J. Young valve NMR tubes. 

### 3.1. NMR Measurements

The NMR spectra were recorded on a Varian VNMRS spectrometer operating at 14.1 T (599.9 MHz for ^1^H and 81.33 MHz for ^17^O) equipped with a 5 mm autoX dual broadband probe and temperature regulation. The sample was left to reach equilibrium at the desired temperature within the magnet for at least 15 min before the NMR measurements. The ^17^O-NMR spectra were recorded with the improved RIDE (ring down elimination) pulse sequence of Kozminsky *et al.* [32] using a 100 μs cawurst adiabatic inversion pulse and the following acquisition parameters: spectral width of about 850 ppm (~69 kHz) centered at 290 ppm, 5 ms relaxation delay, 10 μs (90°) excitation pulse, 10 μs (rof2) preacquisition delay (alfa = 0), 5 ms acquisition time and a number of transients ranging between 5 × 10^4^ and 5 × 10^6^. The receiver ddrtc parameter was optimized to obtain spectra without first-order phase error. The spectra were recorded lock-on without sample spinning. Unless otherwise stated, the processing comprised correction of the first three points of the Free Induction Decay (FID) by backward linear prediction, zero ﬁlling, Fourier transform, zero-order phase correction and baseline correction. The chemical shift scale was calibrated at 25 °C with respect to a sample of pure H_2_O used as an external chemical shift reference (0 ppm).

### 3.2. Computational Details

Structure optimizations of all compounds were performed using the same theory level used in our previous investigations of hypervalent iodine compounds [8,9], which was shown to perform well on these classes of compounds. More specifically the calculations have been carried out at the DFT level employing the PBE0 functional [33], a parameter-free hybrid variant of the Perdew, Burke, and Ernzerhof (PBE) generalized gradient functional [34], as implemented in the commercially available Gaussian 03 suite of program [35]. The effective core-potential valence basis set LANL2DZ (*i.e.*, D95V [36] basis set for the first row elements and the Los Alamos ECP plus DZ on iodine) [37] extended with polarization (d) and diffuse (p) functions [38,39] was employed for all atoms. Numerical integration was performed using a pruned grid having 99 radial shells and 509 angular points per shell. Vibrational analysis was carried out at the same level of theory to check the character of all stationary points. The starting geometries of the minima and TS states of compounds **2**–**4** were constructed by modifying (with appropriate substitutions) the minima and the TS states of compound **1**, as obtained in a previous investigation [8]. The freely available program Molden was used for this purpose [40]. Cartesian coordinates and energies of the optimized geometries of minima and TS are reported in the Supporting Information. NBO version 3 [41] as implemented in Gaussian03 was employed for the analysis of the Wiberg bond index [42]. Graphics of molecular models were generated using the freely available VMD software [43].

## 4. Conclusions

The fluxional behaviour of bis(acetoxy)benzene and some of its derivatives has been studied by ^17^O dynamic NMR and DFT calculations. The activation free energy characterizing the [1,3]-sigmatropic shift of iodine between the oxygen atoms of the carboxylic group is shown to be rather insensitive to the solvent (CDCl_3_, CD_2_Cl_2_, and CD_3_CN), to the substitution of the acetate groups by trifluoroacetate groups and to the presence of electron-donor or electron-acceptor *para-*substituents.

The energy barriers calculated by DFT are in excellent agreement with the activation free energy data determined by NMR. The small or negligible variations with *para-*substitution is in agreement with the invariance of the I-O bond order, which has a rather small value of about 0.4 in all the studied compound. In the transition states, the partial breaking of the I-O bond is accompanied, and thus compensated, by the simultaneous formation of another I-O bond with the carbonylic oxygen atom, and the sum of their bond orders is in all cases between 0.4 and 0.5.

Importantly, this study confirms the recently demonstrated feasibility of ^17^O-Dynamic NMR to obtain quantitative kinetic data on organic molecules without the need of isotopic enrichment.

## Supplementary Materials

Supplementary materials can be accessed at: http://www.mdpi.com/1420-3049/17/11/12718/s1.

## References

[1] Merritt E.A., Olofsson B. (2009). Diaryliodonium salts: A journey from obscurity to fame. Angew. Chem. Int. Ed..

[2] Ladziata U., Zhdankin V.V. (2006). Hypervalent iodine(V) reagents in organic synthesis. ARKIVOC.

[3] Zhu C., Sun C., Wei Y. (2010). Direct oxidative conversion of alcohols, aldehydes and amines into nitriles using hypervalent iodine(III) reagent. Synthesis.

[4] Yusubov M.S., Zhdankin V.V. (2012). Hypervalent iodine reagents and green chemistry. Curr. Org. Synth..

[5] Dohi T., Maruyama A., Minamitsuji Y., Takenaga N., Kita Y. (2007). First hypervalent iodine(III)-catalyzed C-N bond forming reaction: Catalytic spirocyclization of amides to N-fused spirolactams. Chem. Commun..

[6] Zagulyaeva A., Yusubov M.S., Zhdankin V.V. (2010). A general and convenient preparation of [Bis(trifluoroacetoxy)iodo]perfluoroalkanes and [Bis(trifluoroacetoxy)iodo]arenes by oxidation of organic iodides using oxone and trifluoroacetic acid. J. Org. Chem..

[7] Cerioni G., Uccheddu G. (2004). Solution structure of bis(acetoxy)iodoarenes as observed by O-17 NMR spectroscopy. Tetrahedron Lett..

[8] Mocci F., Uccheddu G., Frongia A., Cerioni G. (2007). Solution structure of some lambda(3) iodanes: An O-17 NMR and DFT study. J. Org. Chem..

[9] Fusaro L., Luhmer M., Cerioni G., Mocci F. (2009). On the fluxional behavior of Dess-Martin periodinane: A DFT and O-17 NMR Study. J. Org. Chem..

[10] Fusaro L., Mameli G., Mocci F., Luhmer M., Cerioni G. (2012). Dynamic NMR of low-sensitivity fast-relaxing nuclei: 17O NMR and DFT study of acetoxysilanes. Magn. Reson. Chem..

[11] Lycka A., Holecek J., Handlir K., Pola J., Chvalovsky V. (1986). ^17^O, ^13^C, and ^29^Si NMR spectra of some acyloxy- and diacetoxysilanes and acetoxygermanes. Collect. Czech. Chem. Commun..

[12] Boykin D.W., Baumstark A.L. (1991). ^17^O NMR spectroscopic data for carbonyl compounds. ^17^O NMR Spectroscopy in Organic Chemistry.

[13] Saez P., Rittmann B. (1992). Model-parameter estimation using least-squares. Water Res..

[B14-molecules-17-12718] 14.The errors are standard deviations provided by the analysis of a series of 1000 pseudo-experimental data sets obtained by adding random errors to the experimental LW data as well as to the temperature. These errors were generated from a Gaussian distribution considering a relative standard error on LW of 5% and an absolute standard error on the temperature of ±1 K.

[15] Gerothanassis I.P. (2010). Oxygen-17 NMR spectroscopy: Basic principles and applications (Part I). Prog. Nucl. Magn. Reson. Spectrosc..

[16] Mennucci B., Tomasi J. (1997). Continuum solvation models: A new approach to the problem of solute’s charge distribution and cavity boundaries. J. Chem. Phys..

[17] Cances E., Mennucci B., Tomasi J. (1997). A new integral equation formalism for the polarizable continuum model: Theoretical background and applications to isotropic and anisotropic dielectrics. J. Chem. Phys..

[18] Cossi M., Barone V., Mennucci B., Tomasi J. (1998). Ab initio study of ionic solutions by a polarizable continuum dielectric model. Chem. Phys. Lett..

[19] Cossi M., Scalmani G., Rega N., Barone V. (2002). New developments in the polarizable continuum model for quantum mechanical and classical calculations on molecules in solution. J. Chem. Phys..

[20] Casarini D., Mazzanti A., Lunazzi L. (2010). Recent advances in stereodynamics and conformational analysis by dynamic nmr and theoretical calculations. Eur. J. Org. Chem..

[21] Balakrishnan P., Baumstark A.L., Boykin D.W. (1984). 17O NMR spectroscopy: Unusual substituent effects in para-substituted benzyl alcohols and acetates. Tetrahedron Lett..

[22] Monti D., Orsini F., Ricca G.S. (1986). Oxygen-17 NMR Spectroscopy: Effect of Substituents on Chemical Shifts for o− m− p− Substituted Benzoic Acids, Phenylacetic and Methyl Benzoates. Spectroscopy Lett..

[23] Alcock N.W., Countryman R.M., Esperas S., Sawyer J.F. (1979). Secondary bonding. Part 5. The crystal and molecular structures of phenyliodine(III) diacetate and bis(dichloroacetate). J. Chem. Soc. Dalton Trans..

[24] Kokkou S.C., Cheer C.J. (1986). Structure of diacetato(*m*-tolyl)iodine(III). Acta Cryst..

[25] Pyykko P. (2012). Refitted tetrahedral covalent radii for solids. Phys. Rev. B.

[26] Bondi A. (1964). Van der waals volumes and radii. J. Phys. Chem..

[27] Kotali E., Varvoglis A., Bozopoulos A., Rentzeperis P. (1985). A stable dibenzoiodolyl pyrrolidinedithiocarbamate. J. Chem. Soc. Chem. Commun..

[28] Kotali E., Varvoglis A. (1987). Dialkylcarbamoyl(diaryl)iodanes. J. Chem. Soc. Perkin Trans. I.

[29] Kiprof P. (2005). The nature of iodine oxygen bonds in hypervalent 10-I-3 iodine compounds. ARKIVOC.

[30] Kazmierczak P., Skulski L. (1998). A simple, two-step conversion of various iodoarenes to (Diacetoxyiodo)arenes with Chromium(VI) oxide as the oxidant. Synthesis.

[31] Merkushev E.B., Novikov A.N., Makarchenko S.S., Moskalchuk A.S., Glushova V.V., Kogai T.Y., Polyakova L.G. (1975). Organic compounds of polyvalent iodine. VIII. Simple synthesis of phenyliodosocarboxylates. Zh. Org. Khim..

[32] Kozminski W., Jackowski K. (2000). Application of adiabatic inversion pulses for elimination of baseline distortions in Fourier transform NMR. A natural abundance ^17^O NMR spectrum for gaseous acetone. Magn. Reson. Chem..

[33] Adamo C., Barone V. (1999). Toward reliable density functional methods without adjustable parameters: The PBE0 model. J. Chem. Phys..

[34] Perdew J.P., Burke K., Ernzerhof M. (1996). Generalized gradient approximation made simple. Phys. Rev. Lett..

[35] Frisch M.J., Trucks G.W., Schlegel H.B., Scuseria G.E., Robb M.A., Cheeseman J.R., Montgomery J.A., Vreven T., Kudin K.N., Burant J.C. (2004). Gaussian 03, Revision C.02.

[36] Dunning T.H., Hay P.J., Schaefer H.F. (1976). Modern Theoretical Chemistry: Methods of Electronic Structure Theory.

[37] Wadt W.R., Hay P.J. (1985). Ab initio effective core potentials for molecular calculations. Potentials for main group elements Na to Bi. J. Chem. Phys..

[38] Check C.E., Faust T.O., Bailey J.M., Wright B.J., Gilbert T.M., Sunderlin L.S. (2001). Addition of polarization and diffuse functions to the LANL2DZ basis set for P-Block elements. J. Phys. Chem. A.

[B39-molecules-17-12718] 39.Basis sets were obtained from the Extensible Computational Chemistry Environment Basis Set Database, Version 02/25/04, as developed and distributed by the Molecular Science Computing Facility, Environmental and Molecular Sciences Laboratory, which is part of the Pacific Northwest Laboratory, P.O. Box 999, Richland, Washington 99352, and funded by the U.S. Department of Energy.

[40] Schaftenaar G., Noordik J.H. (2000). The effect of isodensity surface sampling on ESP derived charges and the effect of adding bond-centers on DMA derived charges. J. Comput. Aided Mol. Des..

[41] Weinhold F., Schleyer P.V.R., Allinger N.L., Clark T., Gasteiger J., Kollman P.A., Schaefer H.F., Schreiner P.R. (1998). Natural bond orbital methods. Encyclopedia of Computational Chemistry.

[42] Wiberg K.B. (1968). Application of the pople-santry-segal CNDO method to the cyclopropylcarbinyl and cyclobutyl cation and to bicyclobutane. Tetrahedron.

[43] Humphrey W., Dalke A., Schulten K. (1996). VMD: Visual molecular dynamics. J. Mol. Graph..

