# Supplementary material for: 17O-Dynamic NMR and DFT Investigation of Bis(acyloxy)iodoarenes
Source: Molecules. 2012 Oct 26;17(11):12718–33. doi: 10.3390/molecules171112718 (PMC6268478; doi:10.3390/molecules171112718)
Supplement: Supplementary file 1 [file molecules-17-12718-s001.pdf]

Supporting Information

The [1,3]sigmatopic shift of the iodine between the two oxygen atoms of the acetoxy group(s) is a so-called equally populated two site exchange processes. At the coalescence temperature, T_c, the activation free energy, $\Delta G^{\#}$, can be determined from equation (3), which is obtained using equation (1), *i.e.*, the Eyring equation, and equation (2) for the rate constant of an equally populated two site exchange. *R* is the universal gas constant, *h* the Planck constant, N_A the Avogadro number and Δν is the frequency difference between the coalescing signals (Δν = Δδ × 81.35 for the ^{17}O-NMR spectra recorded in the present work):

$$k_c = \frac{RT_c}{hN_A} \cdot exp\left(\frac{-\Delta G^{\#}}{RT_c}\right) \tag{1}$$

$$k_c = \frac{\pi \Delta \upsilon}{\sqrt{2}} \tag{2}$$

$$\Delta G = RT_c\left(ln\frac{R\sqrt{2}}{h\pi N_A} + ln\frac{T_c}{\Delta \nu}\right) = RT_c\left(22.96 + ln\frac{T_c}{\Delta \nu}\right) \tag{3}$$

Figure S1. ^{17}O-NMR spectra recorded for bis(acetoxy)iodobenzene dissolved in $CDCl_3$ at 14.1T (1 ppm = 81.356 Hz) and −30 °C (left) or −15 °C (right): (**a**) Full spectra obtained after Fourier Transform and zero-order phase correction *(no apodization and no backward linear prediction of the FID, no first-order phase correction and no baseline correction of the spectrum)*. (**b**) Fittings using two Lorenztian lines and no constraint. (**c**) Fittings using two Lorenztian lines of identical integrated intensity.

(a)

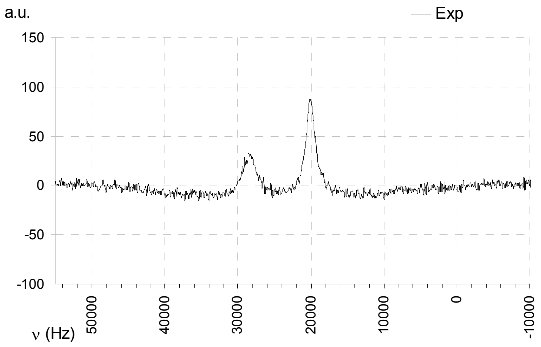

(T = −30 °C, 1.5×10^6 scans (transients))

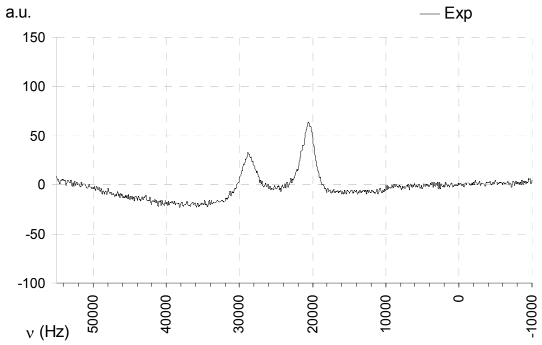

(T = −15 °C, 4.5×10^6 scans (transients))

(b)

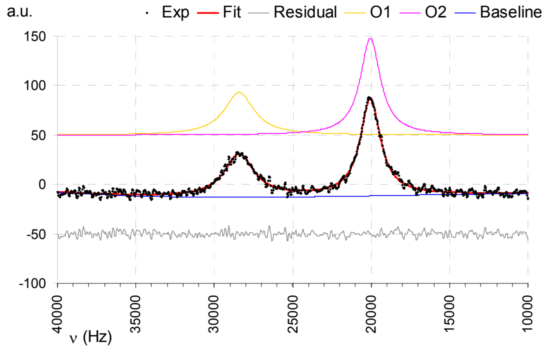

(T = −30 °C, Integral ratio O1/O2: 0.65)

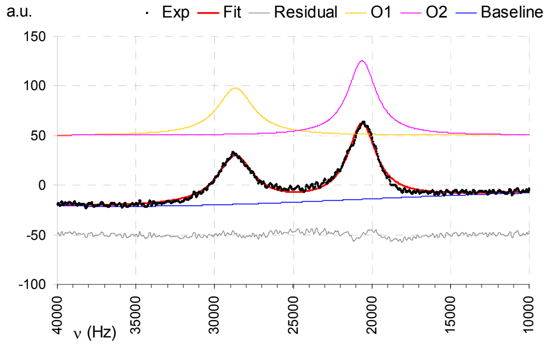

(T = −15 °C, Integral ratio O1/O2: 0.81)

Figure S1. *Cont.*

(c)

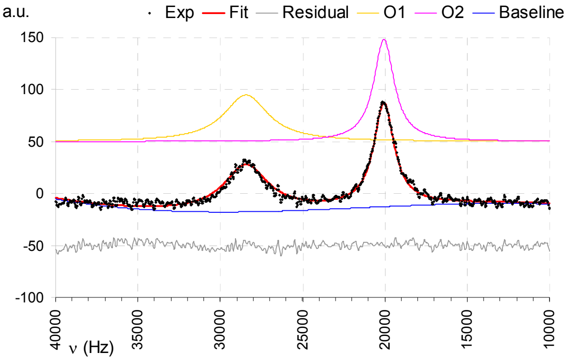

(T = −30 °C, Integral ratio O1/O2 constrained to 1)

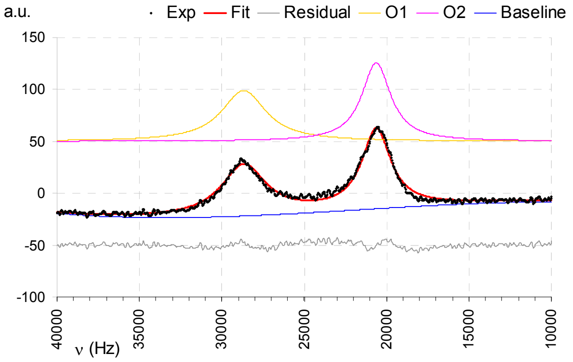

(T = −15 °C, Integral ratio O1/O2 constrained to 1)

Figure S2. ^{17}O-NMR spectra recorded at 12.5 °C and 14.1 T (**A**) for compound **3** dissolved in $CDCl_3$ and (**B**) for compound **4** dissolved in a 98/2 (v/v) mixture of $CDCl_3$/DMSO-d_6. The processing comprised correction on the first 3 points of the Free Induction Decay (FID) by backward linear prediction, exponential multiplication of the FID with a line broadening factor of 100 Hz, Fourier transform, zero-order phase correction, and baseline correction.

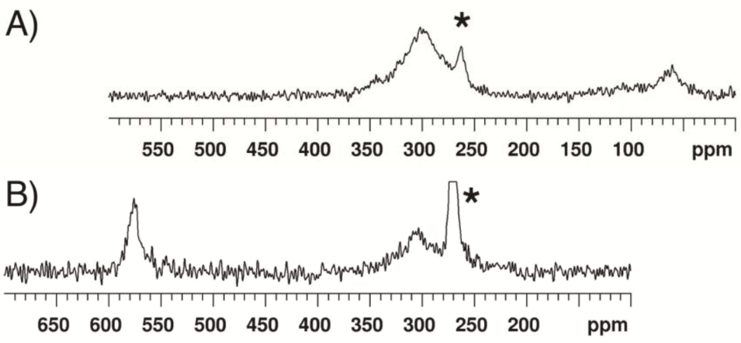

Figure S3. Optimized Pbe0/LANL2DZDP of transition states in the [1,3] sigmatropic shift of iodine between the acetoxy oxygen in compounds **2**–**4**. Two representations are given for each compound; they are rotated by approximately 90° around the I-O bonds. Color code: cyan = carbon, white = hydrogen, red = oxygen, green = fluorine, pink = iodine, blue = nitrogen.

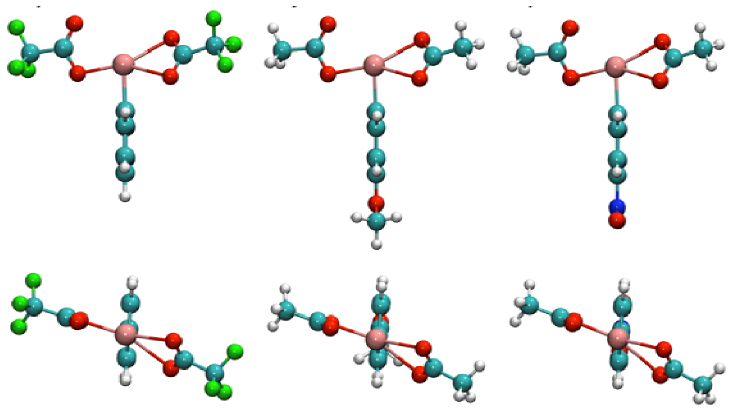

Table S1. Cartesian coordinates of PBE0/LANL2DZDP global and relative minimum energy geometries of compounds **2.** The notation used to indicate the conformers is the same as in Figure 9.

Conformer A

C	-0.003508	0.000018	-0.002097
C	-0.002519	-0.064022	1.393726
C	1.203962	-0.064666	2.098683
C	2.390632	0.009057	1.370061
C	2.419071	0.078407	-0.022381
C	1.200984	0.068609	-0.707020
I	4.210938	0.015929	2.413338
O	3.338682	1.756728	3.357910
C	4.170982	2.252069	4.231044
C	3.571829	3.496169	4.946679
F	3.274590	4.448862	4.056679
O	4.594886	-1.726795	1.189033
C	5.771976	-2.214248	1.467057
C	6.093539	-3.459571	0.592603
F	7.289011	-3.950189	0.887348
O	6.556327	-1.787643	2.290375
F	6.079001	-3.129435	-0.704996
F	5.180930	-4.416869	0.789584
O	5.280386	1.832815	4.492771
F	4.427741	3.994705	5.827366
F	2.447004	3.161437	5.591419
H	-0.942065	-0.118052	1.943683
H	1.209571	-0.110967	3.186278
H	3.360051	0.128282	-0.567455
H	1.200289	0.119089	-1.795859
H	-0.949269	-0.003553	-0.544142

Conformer B

C	-0.161040	-0.307483	-0.086213
C	0.038338	-0.928081	1.150427
C	1.260929	-0.797326	1.813070
C	2.260709	-0.037693	1.202486
C	2.089613	0.593346	-0.030349
C	0.856937	0.449345	-0.672699
I	4.097777	0.151698	2.181682
O	3.388287	1.963558	3.091601
C	3.363982	3.034933	2.338426
C	2.892380	4.267473	3.165628
F	1.658181	4.062447	3.641212
O	4.494672	-1.754193	1.240544
C	5.683606	-2.173219	1.570735
C	6.005100	-3.549640	0.921635
F	7.221446	-3.958283	1.255930
O	6.478868	-1.603649	2.292355
F	5.937307	-3.464673	-0.411135
F	5.122554	-4.470272	1.327428
O	3.642592	3.131386	1.163861
F	2.874303	5.361761	2.414938
F	3.710149	4.479335	4.201808
H	-0.757400	-1.517073	1.606423
H	1.424423	-1.278228	2.776170
H	2.882829	1.195000	-0.469827
H	0.697623	0.935308	-1.635216
H	-1.118802	-0.413471	-0.596001

Conformer C

C	-0.366305	-0.001601	-0.210205
C	-0.269510	-0.661104	1.018497
C	0.936072	-0.664215	1.724599
C	2.021223	0.007628	1.158320
C	1.957533	0.675031	-0.066473
C	0.738997	0.662549	-0.749869
I	3.836489	0.014648	2.198829
O	3.272934	1.875862	3.100093
C	3.380846	2.953645	2.362981
C	3.010127	4.216572	3.195605
F	1.750885	4.129180	3.638908
O	4.340206	-1.846791	1.263260
C	3.764894	-2.926370	1.732433
C	4.303345	-4.188650	0.995956
F	3.730473	-5.289917	1.465409
O	2.926763	-3.011447	2.602203
F	5.626154	-4.298124	1.159913
F	4.049094	-4.107045	-0.314976
O	3.707425	3.036760	1.199951
F	3.119424	5.316062	2.460396
F	3.819386	4.334205	4.253884
H	-1.132291	-1.180659	1.435205
H	1.030556	-1.190861	2.672149
H	2.819839	1.205374	-0.465594
H	0.659511	1.178462	-1.706681
H	-1.312139	-0.005255	-0.752352

Conformer D

C	-0.377404	-0.130101	0.014969
C	-0.120762	-0.711810	1.260079
C	1.146725	-0.603957	1.836872
C	2.127806	0.095907	1.131023
C	1.902961	0.687276	-0.112642
C	0.626040	0.564148	-0.666655
I	4.027684	0.261004	1.989182
O	3.357303	1.997579	3.049436
C	3.220844	3.102179	2.356287
C	2.783993	4.272103	3.286571
F	1.610807	3.992052	3.866888
O	4.547023	-1.623188	1.111966
C	4.777772	-1.635207	-0.178687
C	5.194878	-3.063292	-0.638686
F	5.440734	-3.087472	-1.942353
O	4.692514	-0.716877	-0.961852
F	4.217985	-3.940256	-0.377555
F	6.296956	-3.461406	0.006375
O	3.383986	3.264186	1.168405
F	2.651618	5.399164	2.598963
F	3.690203	4.469395	4.250419
H	-0.905416	-1.252560	1.789111
H	1.357594	-1.053061	2.805982
H	2.693825	1.224431	-0.631139
H	0.421995	1.016221	-1.637108
H	-1.369549	-0.219058	-0.428057

Table S2. Cartesian coordinates of PBE0/LANL2DZDP global and relative minimum energy geometries of compounds **3**. The notation used to indicate the conformers is the same as in Figure 9.

Conformer A

C	0.004791	-0.000564	-0.002669
C	0.005230	-0.006525	1.399803
C	1.223482	-0.009780	2.100890
C	2.428609	-0.007149	1.410936
C	2.419544	-0.001182	0.011200
C	1.219224	0.002118	-0.694498
O	-1.110450	-0.009509	2.163487
C	-2.362448	-0.006473	1.506794
I	4.226643	0.002798	-1.031013
O	3.967518	-2.148725	-0.894147
C	4.996255	-2.719420	-1.485037
O	5.901816	-2.070590	-2.001942
O	3.971558	2.153298	-0.870538
C	5.000985	2.728418	-1.455733
C	4.962783	4.235987	-1.417169
O	5.904992	2.083503	-1.980347
C	4.953585	-4.227183	-1.465034
H	1.213389	0.006765	-1.783966
H	-0.925449	0.002051	-0.566376
H	1.200425	-0.014402	3.190118
H	3.367646	-0.009719	1.963133
H	5.838983	-4.628728	-1.966040
H	4.912349	-4.581930	-0.427477
H	4.043373	-4.576785	-1.968569
H	4.043032	4.595208	-1.895721
H	5.839017	4.641194	-1.931224
H	4.945874	4.577118	-0.374271
H	-3.118661	-0.009695	2.297208
H	-2.488905	0.894357	0.887076
H	-2.489247	-0.901943	0.879425

Conformer B

C	-0.025413	0.004126	0.021501
C	-0.009749	0.003649	1.418510
C	1.209340	-0.000184	2.092881
C	2.426336	-0.000392	1.400469
C	2.407677	0.002589	0.011982
C	1.186918	0.002427	-0.685032
I	1.208683	-0.024556	4.176337
O	1.445344	2.105574	4.180759
C	2.664249	2.573822	3.938833
O	3.620329	1.878889	3.629895
O	0.830454	-2.131766	3.878997
C	0.715173	-2.712388	5.056432
C	0.466117	-4.197837	4.977714
O	0.807351	-2.092432	6.112555
C	2.735000	4.076124	4.082010
H	3.371402	0.023380	1.939875
H	3.335856	0.007141	-0.558424
O	1.281910	0.003745	-2.033244
H	-0.981519	0.006001	-0.497045
H	-0.951357	0.002284	1.966162
H	-0.431343	-4.393961	4.377793
H	1.311285	-4.684023	4.473889
H	0.345202	-4.606109	5.985191
H	2.033374	4.546060	3.381039
H	3.754036	4.419343	3.881154
H	2.429610	4.366818	5.095050
C	0.083154	0.004096	-2.782728
H	0.383965	0.003475	-3.834451
H	-0.519580	-0.893602	-2.576951
H	-0.518536	0.902731	-2.577849

Conformer C

C	-0.096029	-0.019736	0.014902
C	-0.071277	0.002054	1.408106
C	1.130025	0.019583	2.128313
C	2.326841	0.013624	1.424687
C	2.326778	-0.007192	0.018562
C	1.112882	-0.023737	-0.685261
I	-1.876820	0.011335	2.447635
O	-2.010917	2.124503	2.152600
C	-2.422102	2.549907	0.963799
O	-2.649399	1.820996	0.010044
O	-1.704276	-2.100614	2.719816
C	-0.888644	-2.525819	3.678553
C	-0.934366	-4.027544	3.835966
O	-0.177245	-1.796921	4.352571
C	-2.570127	4.052750	0.920340
H	1.127071	0.009770	3.216688
H	3.281928	0.022296	1.948709
O	3.545041	-0.009691	-0.566062
H	1.090337	-0.035531	-1.772609
H	-1.041363	-0.005364	-0.524565
H	-1.962916	-4.350170	4.040395
H	-0.622710	-4.503433	2.897326
H	-0.271609	-4.334452	4.650280
H	-3.266005	4.381620	1.702260
H	-2.933471	4.360685	-0.064422
H	-1.599958	4.522202	1.127754
C	3.602936	-0.032007	-1.978877
H	4.665752	-0.031528	-2.237810
H	3.130135	-0.938714	-2.385843
H	3.122273	0.857039	-2.414538

Conformer D

C	-0.109042	0.047816	0.023019
C	-0.080795	-0.013955	1.414417
C	1.122561	-0.073621	2.127560
C	2.314764	-0.070501	1.417055
C	2.309849	-0.008643	0.012224
C	1.094383	0.050684	-0.686905
I	-1.883154	-0.017284	2.454345
O	-1.886674	2.118620	2.406503
C	-1.047678	2.735782	3.233721
O	-0.250115	2.160725	3.955812
O	-1.987091	-2.138106	2.218474
C	-1.179777	-2.864515	2.986445
C	-1.391972	-4.347428	2.789558
O	-0.357792	-2.393434	3.754948
C	-1.189748	4.238606	3.169007
H	1.121382	-0.121288	3.213862
H	3.271707	-0.115835	1.935551
O	3.525689	-0.011435	-0.577646
H	1.068157	0.099005	-1.773140
H	-1.053900	0.093773	-0.516775
H	-2.441692	-4.603499	2.980024
H	-1.173697	-4.613477	1.747257
H	-0.736681	-4.906703	3.463611
H	-0.957549	4.585126	2.153712
H	-0.510048	4.704856	3.888147
H	-2.226610	4.525767	3.383992
C	3.579349	0.049290	-1.988854
H	4.641039	0.035866	-2.252045
H	3.080676	-0.818094	-2.447713
H	3.122899	0.976007	-2.368894

Table S3. Cartesian coordinates of PBE0/LANL2DZDP global and relative minimum energy geometries of compounds **4**. The notation used to indicate the conformers is the same as in Figure 9.

Conformer A

C	0.000055	-0.019879	-0.001618
C	0.000812	-0.020081	1.392533
C	1.222081	-0.000014	2.062784
C	2.442416	0.021283	1.390873
C	2.441235	0.023636	-0.003275
C	1.220173	0.002499	-0.680938
N	1.223104	-0.001362	3.534720
O	0.137854	-0.022722	4.100309
I	1.218704	0.004434	-2.792276
O	2.716255	-1.523808	-2.463248
C	3.103655	-1.944108	-3.652443
C	4.154771	-3.022280	-3.612615
O	-0.278411	1.532044	-2.458359
C	-0.667431	1.954554	-3.646241
O	-0.200054	1.493327	-4.683402
O	2.634861	-1.480959	-4.688107
C	-1.718510	3.032630	-3.602977
O	2.309140	0.018966	4.098837
H	-0.942318	-0.024009	-0.545055
H	-0.926744	-0.038354	1.960795
H	3.370761	0.038517	1.957877
H	3.382851	0.028763	-0.548014
H	4.385752	-3.348981	-4.630320
H	5.060500	-2.634659	-3.128755
H	3.796534	-3.868065	-3.012768
H	-2.623567	2.644100	-3.118587
H	-1.950895	3.361205	-4.619758
H	-1.359462	3.877313	-3.002063

Conformer B

C	0.021515	-0.263204	-0.061961
C	0.070697	-0.315646	1.329977
C	1.271334	-0.006234	1.973909
C	2.424897	0.355801	1.276398
C	2.371726	0.410296	-0.117051
C	1.174028	0.099761	-0.757063
I	1.329765	-0.109763	4.070940
O	1.230555	2.022171	4.126943
C	2.365644	2.689220	3.934437
O	3.417307	2.166601	3.599043
O	1.261429	-2.216095	3.629026
C	1.267003	-2.873068	4.774439
C	1.237016	-4.370793	4.619147
O	1.297695	-2.295209	5.857194
C	2.201971	4.170381	4.172036
H	3.339797	0.610426	1.808530
H	3.243380	0.687406	-0.706228
N	1.121296	0.156885	-2.228714
H	-0.889361	-0.495747	-0.609460
H	-0.813982	-0.599643	1.897322
H	0.357653	-4.666932	4.033678
H	2.126394	-4.698938	4.066248
H	1.212430	-4.843183	5.605143
H	1.415145	4.566041	3.517499
H	3.148658	4.682622	3.978141
H	1.883428	4.343741	5.207769
O	0.056560	-0.118293	-2.765761
O	2.144892	0.475479	-2.818043

Conformer C

C	-0.038305	-0.010160	0.024319
C	-0.036600	0.000142	1.420976
C	1.134915	0.009524	2.181386
C	2.358196	0.009083	1.511143
C	2.359971	-0.001746	0.117508
C	1.189102	-0.011650	-0.638338
I	-1.880426	0.001579	2.423810
O	-1.970061	2.104057	2.114777
C	-2.362046	2.527117	0.914415
O	-2.566374	1.789848	-0.036780
O	-1.672249	-2.100993	2.668331
C	-0.878186	-2.524386	3.650019
C	-0.922140	-4.024417	3.806678
O	-0.189892	-1.787509	4.338022
C	-2.515657	4.027299	0.865165
H	1.095789	-0.009387	3.268725
H	3.301130	0.014952	2.053696
N	3.655127	-0.002767	-0.586913
H	1.246064	-0.018307	-1.724723
H	-0.972315	0.009522	-0.533742
H	-1.951235	-4.346150	4.010077
H	-0.609920	-4.501657	2.869075
H	-0.261573	-4.329855	4.623087
H	-3.245511	4.350358	1.618072
H	-2.841375	4.332473	-0.133304
H	-1.558439	4.503589	1.112583
O	3.631789	-0.018691	-1.810128
O	4.669298	0.012372	0.097399

Conformer D

C	-0.039809	0.061203	0.026891
C	-0.046071	-0.004214	1.422071
C	1.120872	-0.064477	2.184638
C	2.345636	-0.058754	1.516933
C	2.353718	0.006481	0.124802
C	1.187051	0.066625	-0.635418
I	-1.897305	-0.011736	2.405244
O	-1.862840	2.113244	2.341200
C	-1.037887	2.722407	3.193259
O	-0.261936	2.132000	3.925443
O	-1.951673	-2.120770	2.141163
C	-1.154518	-2.841494	2.930346
C	-1.350242	-4.324660	2.732880
O	-0.356048	-2.355672	3.713394
C	-1.171110	4.224465	3.136981
H	1.075121	-0.114475	3.269545
H	3.285991	-0.104062	2.061964
N	3.650477	0.012279	-0.576577
H	1.249455	0.116293	-1.720299
H	-0.968429	0.107246	-0.539367
H	-2.396979	-4.590534	2.926593
H	-1.132819	-4.589424	1.690234
H	-0.688488	-4.876362	3.406567
H	-0.940767	4.577104	2.123536
H	-0.488218	4.682022	3.858401
H	-2.206209	4.514364	3.356985
O	3.629857	0.070189	-1.799093
O	4.662633	-0.041124	0.107941

Table S4. Cartesian coordinates of PBE0/LANL2DZDP optimized structure of the transition state in the [1,3] sigmatropic shift of iodine between the acetoxy oxygen in compounds **2–4**.

Compound **2**

C	0.000000	0.000000	0.000000
C	0.000000	0.000000	1.396635
C	1.205823	0.000000	2.106262
C	2.424324	0.000565	1.426293
C	2.395765	0.012895	0.031684
C	1.209189	0.002181	-0.702012
I	4.193144	0.104807	-1.049742
O	5.237758	-2.047960	-1.582422
C	5.026451	-2.570315	-0.457589
C	5.617753	-3.990382	-0.238975
O	3.612188	2.179113	-1.251423
C	4.508473	2.800707	-1.965810
C	4.141723	4.302219	-2.153447
O	5.514063	2.319674	-2.447491
O	4.395809	-2.023885	0.464416
H	1.217551	0.002422	-1.791134
H	-0.939794	-0.005356	-0.552107
H	-0.946227	-0.005449	1.937971
H	1.202478	-0.006064	3.196261
H	3.367215	-0.021394	1.968848
F	5.176386	-4.823709	-1.184714
F	6.951694	-3.941679	-0.308575
F	5.280176	-4.488446	0.946734
F	2.966527	4.414908	-2.781536
F	5.064884	4.925419	-2.871117
F	4.043635	4.908312	-0.966507

Compound **3**

C	0.000000	0.000000	0.000000
C	0.000000	0.000000	1.404809
C	1.215944	0.000000	2.106759
C	2.422330	0.002318	1.405204
C	2.406313	0.016520	0.012960
C	1.201332	0.004262	-0.698327
I	4.192382	0.100067	-1.078057
O	5.256532	-2.027715	-1.517326
C	5.118518	-2.529119	-0.356034
C	5.750647	-3.873210	-0.080784
O	3.561002	2.156082	-1.353473
C	4.463708	2.746732	-2.108299
C	4.161820	4.198727	-2.386832
O	5.454240	2.162238	-2.537969
O	4.482757	-1.921831	0.539448
H	1.195222	0.005053	-1.787814
H	-0.954607	-0.007306	-0.524898
H	1.238119	-0.010562	3.194224
H	3.365350	-0.023915	1.947694
H	5.441699	-4.587493	-0.853265
H	6.841652	-3.770764	-0.144687
H	5.469931	-4.237891	0.911188
H	3.185714	4.284746	-2.880340
H	4.945251	4.621518	-3.021999
H	4.102875	4.750090	-1.440084
O	-1.218132	-0.006814	1.991571
C	-1.273406	-0.017848	3.404333
H	-2.335778	-0.024015	3.665222
H	-0.792538	-0.917216	3.818138
H	-0.799772	0.878847	3.832197

Compound **4**

C	0.000000	0.000000	0.000000
C	0.000000	0.000000	1.392624
C	1.175298	0.000000	2.143966
C	2.398422	-0.001140	1.477658
C	2.402589	0.012522	0.082478
C	1.225528	0.005135	-0.667119
I	4.229178	0.106724	-0.981973
O	5.335887	-1.970655	-1.302110
C	5.131848	-2.485116	-0.152434
C	5.780124	-3.811597	0.156585
O	3.505876	2.127969	-1.269431
C	4.404832	2.739869	-2.013581
C	4.074117	4.179584	-2.311752
O	5.417391	2.172263	-2.415295
O	4.420087	-1.895409	0.693637
H	1.253091	0.009923	-1.755797
H	-0.943773	-0.005572	-0.541076
H	1.120611	-0.005052	3.230501
H	3.330814	-0.027244	2.036932
H	5.564230	-4.522220	-0.649852
H	6.868451	-3.672421	0.190826
H	5.426150	-4.199726	1.115447
H	3.090470	4.242359	-2.793187
H	4.843292	4.606218	-2.961324
H	4.018592	4.743076	-1.371817
N	-1.291838	-0.003622	2.098860
O	-1.266752	0.004569	3.322400
O	-2.308460	-0.013565	1.417105

Imaginary frequencies ν (cm-1)

Compound **2**: -179.8

Compound **3**: -189.5

Compound **4**: -183.2
